# Case report of old anterior dislocation of the shoulder joint and review of the literature

**DOI:** 10.3389/fsurg.2022.994204

**Published:** 2022-11-11

**Authors:** Weiming Guo, Ming Hui, Yuan Yang, Teng Wan, Guang-Gui Zeng, Peiguan Huang, Xiaoxu Wang, Mingjun Qiu

**Affiliations:** ^1^ Sports Medicine Department, Huazhong University of Science and Technology Union Shenzhen Hospital; the 6th Affiliated Hospital of Shenzhen University Health Science Center, Shenzhen, China; ^2^ The Second Affiliated Hospital, Department of Orthopaedic Surgery, Hengyang Medical College, University of South China, Hengyang, Hunan, China; ^3^ Hengyang Medical College, University of South China, Hengyang, China

**Keywords:** anterior shoulder dislocation, arthroscopy, traumatic, surgery, case

## Abstract

**Background:**

Dislocation of the shoulder joint is the most common type of joint dislocation. It is rare to be in a persistent dislocation that has not been reset. Successful arthroscopic treatment of the obsolete shoulder is relatively uncommon.

**Case report:**

We report a rare case of persistent anterior dislocation of the old shoulder joint in a 30-year-old female patient. The patient underwent an emergency shoulder dislocation at a local hospital after a traumatic injury and re-dislocated persistently after surgery. 26 days later, she was admitted to our department for treatment, mainly because of joint deformity and limitation of motion. We adopted arthroscopic release and repositioning surgery. The patient was followed up for 1 year after surgery. Functional recovery was satisfactory.

**Conclusion:**

The state of obsolete shoulder dislocation rarely occurs after shoulder dislocation and the prognosis of the patient is good after complete arthroscopic release and repositioning. It provides a reference for clinical arthroscopic treatment of old shoulder dislocations.

## Introduction

The shoulder joint mainly includes the glenohumeral, acromioclavicular, sternoclavicular and scapulothoracic brachial joints; and shoulder dislocation mainly refers to the glenohumeral joint and is the most common joint dislocation seen by orthopaedic surgeons in emergency situations, reported to occur in 8.2–23.9 per 100,000 people per year (1–4). And anterior dislocations account for 98% of all shoulder dislocations (5, 6). Shoulder dislocations are more common in younger age groups who are active in sports and daily activities (7). The diagnosis of acute dislocation is uncomplicated in patients who have obvious symptoms associated with the dislocated joint with significant deformity and are able to be seen promptly. However, patient negligence in seeking post-traumatic care and patient failure to present promptly result in rare old undisplaced shoulder dislocations. They are often difficult to reposition by closed resetting and usually require surgical intervention to reposition and stabilise the joint. The treatment options for old anterior shoulder dislocations are unclear (8). In the case described here, a patient with a traumatic old unresolved anterior shoulder dislocation was presented. The patient did not receive further treatment as multiple resetting after the injury had failed, however, prolonged pain, deformity and dysfunction led to admission to our hospital for further consultation. We therefore report this and review the relevant literature.

## Case reports

**Figure 1:**
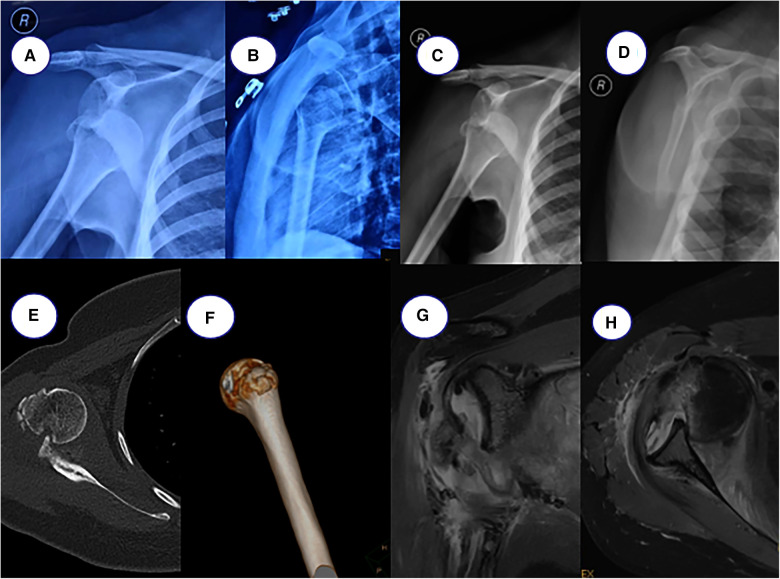
(**A,B**) postoperative x-ray after external emergency repositioning, (**C,D**) preoperative x-ray, (**E,F**) preoperative CT film, (**G,H**) preoperative MRI.

**Figure 2:**
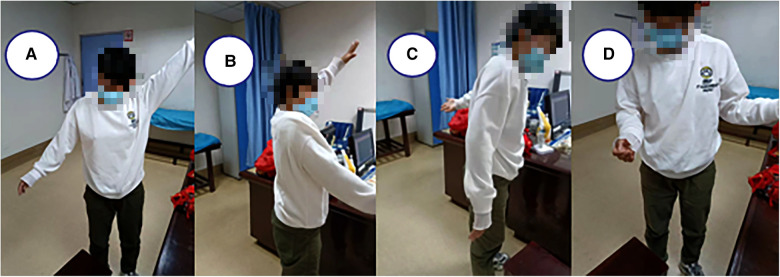
Preoperative physical examination for shoulder joint mobility in all directions.

A 30-year-old female patient complained of right shoulder deformity and pain with limited movement for 26 days, and it affected his appearance. The patient suffered a painful deformity and limited movement of the right shoulder as a result of a motorcycle fall 26 days ago. He was admitted to the local hospital as an emergency. x-ray showed anterior dislocation of the right shoulder joint and fracture of the greater tuberosity of the humerus. Totally, he was admitted to the local hospital for three times of intermittent and consecutive manipulation and repositioning of the right shoulder joint (Figures 1A,B). The patient was in good health with no history of chronic disease. Physical examination found a square shoulder deformity, subacromial hollowing and slight pressure pain on the lateral aspect of the shoulder peak. Joint mobility was examined with active abduction of up to 45°, anterior flexion of about 60°, posterior extension of about 5°, lateral external rotation of about 0°, and internal rotation flat at the level of the greater tuberosity. Passive activity range is the same as active. The patient had a flexible fixed shoulder joint, Dugas sign (+) and could not cooperate with special examinations. The preoperative VAS score was 4 and the ASES score was 7 (Figure 2). Laboratory tests revealed no abnormalities. x-ray showed anterior subluxation of the right humeral head and fracture of the greater tuberosity of the humerus (Figures 1C,D). CT showed additional Hill-Sachs injury of the humeral head (Figures 1E,F). MRI showed continuous supraspinatus and infraspinatus tendons and injury of the anterior inferior glenoid lip (Figures 1G,H). The neuromyogram was normal. Vascular ultrasound of the upper extremity showed no abnormalities.

In view of the rare clinical reports of old shoulder dislocations, there is no uniform treatment plan. After preoperative discussion in the whole department, a decision was made to adopt limited duration surgical treatment (Table 1). After general anesthesia, manual repositioning was attempted, and minimally invasive arthroscopic treatment was adopted after repositioning failed. The joint was released in the order of intra-articular-subacromial-intra-articular. Intraoperatively, scar tissue filling of the humeral glenoid joint was seen and the shoulder joint was unsuccessfully repositioned after cleaning.

**Table 1:** Pre-operative discussion options.

Period of surgery	a. limited-term surgery? b. elective surgery
Surgical approach	a. Minimally invasive treatment attempted first? b. Minimally invasive failure and incisional repositioning?
How to loosen and reset	a. Why did all 3 resetting attempts fail? b. What are the factors that prevent reset? c. Release sequence: intra-articular? Subacromial space? Other?
How Hill-Sachs injuries are treated.	a. Sub-ganglionic tendon filling? b. Bone grafting?
Are large nodules fixed.	a. Reset and fixation? b. Conservative treatment?
How to stabilise the joint after release.	a. Anchor nailing for joint capsule repair? b. Clinically fixed? c. Soft tissue balance?

**Figure 3:**
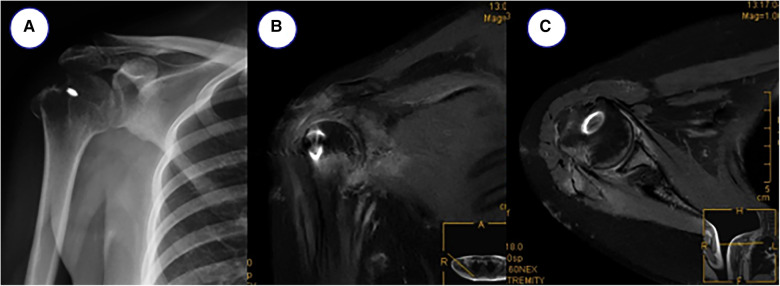
Postoperative reexamination. (**A**) x-ray orthopantomogram, (**B,C**) MRI images.

The posterior defect of the humeral head was seen to be embedded with the anterior glenoid, then the subacromial crest was entered arthroscopically and the subacromial bursa tissue was removed, the supraspinatus and infraspinatus tendons were continuous and in satisfactory tension, the greater humeral tuberosity was stable and no further treatment was undertaken. The rotator cuff gap and subscapularis were then released subacromially, and the humeral head was gradually repositioned after satisfactory release under anterior surveillance. The arthroscope was reentered into the joint cavity, the subscapularis muscle was released posteriorly, the injured glenoid lip was repaired and the humeral head was repositioned to the centre of the articular glenoid without external force. the Hill-Sachs injury was filled with the infraspinatus tendon. The final anterior and posterior rotator cuff tension of the shoulder was satisfactory and the shoulder was fixed with a postoperative abduction pack. The postoperative rehabilitation program included brace-assisted immobilization on the second day after surgery and the initiation of passive functional exercises, including anterior flexion and external rotation. 6 weeks later the brace was removed and active functional exercises were initiated. Regular outpatient follow-up. At 1 year post-operative follow-up, the right shoulder joint showed no difference in morphology from the contralateral shoulder joint, with activity levels: abduction 90°, adduction 30°, anterior flexion 85°, posterior extension 30°, external rotation 60° and internal rotation 65°, which were essentially normal. VAS score was 0 and ASES score was 9 (Figure 3). The patient was also informed of the study and he agreed to publish his treatment.

## Discussion

Shoulder dislocations are relatively common in clinical joint dislocations. There is no unified standard consensus on the management of shoulder dislocations in all age groups: whether shoulder dislocations should be treated conservatively or surgically is still under discussion. Manual repositioning under emergency care is routinely recommended. There is no consensus on the method of repositioning, with Kocher's method recommended, foot stirrups not recommended, and brachial plexus anaesthesia or intra-articular local anaesthesia for repositioning in elderly patients with osteoporosis. There is also no agreement on the type of braking and there is still discussion on whether to immobilise the shoulder joint in internal or external rotation (9–11). A study has reported no difference between these two types of braking in terms of recurrence rates and healing outcomes of joint dislocation and return to activity levels (12). Negligent and untimely access to medical care after trauma results in rare old undisplaced shoulder dislocations that are challenging to treat, and the patients in this paper were primarily untimely for further consultation. The success rate of resetting under general anaesthesia for old shoulder dislocations is low (40%) because the risk of resetting is greatly increased the longer the joint is not resetting under emergency care following traumatic dislocation, contracture of the joint capsule, development of surrounding scar tissue, entrapment of surrounding injured tissue and formation of fibrotic soft tissue (13).

Also related literature has found many terms and durations used to describe obsolete shoulder dislocations. While most authors define a delayed diagnosis of shoulder dislocation of more than 3 weeks as obsolete shoulder dislocation, some experts consider the cut-off point for the division between acute and obsolete cases to be 24 h after to 1 month (14). In addition, there are various terms used to describe this condition, including chronic, unrecognised, missed, old unreset, chronic unreduced and old dislocation (14). Although the relevant literature expresses the same concept, these terms may cause some confusion within the specialty; and in order to understand the disease itself, they are not understood as the same concept within the relevant specialty books and in the clinic, posing difficulties for article searching and writing, and they are not the same as each other. Therefore, they need to be standardised to avoid long-term confusion. Strategies for managing this issue are also controversial and remain a challenge. The patient in this article has been unsuccessfully diagnosed and treated since the injury and has remained in dislocation. We therefore refer to this as an obsolete non-repositioning.

At present, there is no uniform protocol for treatment, based on this article in that treatment is experimental or more empirical than evidence-based. Of course, with the development of multimedia, a combination of relevant specialist books and literature is available on the various methods of treatment of old dislocations. These include conservative treatment, resetting, incisional repositioning and resection arthroplasty, as well as the development of minimally invasive techniques, arthroscopic repositioning and related repair methods. In this paper, however, the patient first attempted a manipulative resurfacing even after general anaesthesia, which ultimately failed. Although the literature suggests that attempts at resetting should be abandoned if the dislocation is more than 6 weeks old. If resetting fails, the next step is surgical repositioning (8). Because of the extensive fibrosis in a long-dislocated shoulder joint, adequate release of the joint capsule, excision of the entrapment and scar tissue and balancing of the soft tissue tension of the joint are required to reposition the joint; otherwise, the abnormal soft tissue blocks repositioning of the joint and at the same time the soft tissue tension is not balanced and the stability of the joint cannot be maintained after repositioning. In addition, scar tissue and laxity covering the surface of the pelvis must be removed prior to repositioning. For this, we take an arthroscopic, minimally invasive release and reposition. On entering the humeral glenoid joint, the humeral head could not be seen within the joint due to the large amount of scar tissue covering the surrounding area, so the joint was cleared of scar tissue. The long head of the biceps tendon was dislocated and seized. The joint capsule was released after severing the long head tendon, but was found to be difficult to reset. The subacromial crest was then entered and explored to see the rostroscapular ligament entrapment, so the rostroscapular ligament was cleared. The glenohumeral joint was then accessed to release the anterior joint capsule and gradually reposition the humeral glenoid joint.

Depending on the humeral head bone defect, the Hill-Sachs injury was filled using the infraspinatus tendon. For stabilisation of the joint after release, most reports recommend a direct approach of internal fixation through the shoulder joint after repositioning. Neviaser recommended screw fixation for 3–4 weeks and Wilson and Mckeever recommended steel pin fixation between the acromioclavicular humerus (15, 16). Goga reported on a group of 10 cases with incisional repositioning across the acromioclavicular humeral pin fixation for 4 weeks, with three excellent, five good and two fair results according to the Rowe and Zarin system (14). However, consider that internal fixation additionally increases damage to the humeral head and articular glenoid and tends to lead to joint adhesions and stiffness. If early shoulder movement improves the nutrition of the articular cartilage and reduces damage to the articular surface. In our case, the Hill-Sach injury was small, the greater humeral tuberosity had partially healed and the dislocation time was relatively short. In our case, the anterior shoulder contracture was extensively released and the posterior Hill-Sach injury was repaired by filling the sub-gonadal tendon to enhance the soft tissue balance anteriorly and posteriorly, with a short period of early abduction brace fixation, which is more effective than internal fixation with a metal object. This allows intermittent passive movement within a safe range several times a day, morning, noon and night, without fear of re-dislocation and to avoid stiffness.

The most common injuries accompanying old anterior shoulder dislocations are Bankart and Hill-Sachs injuries, with Bankart injuries referring to injuries to the anterior aspect of the joint capsule and the labrum of the glenoid and Hill-Sachs injuries referring to injuries to the posterior lateral aspect of the humeral head, with a reported correlation of 90%–97% and CT and MRI examinations emerging as the gold standard for their diagnosis (17, 18). The advantage of arthroscopic management is that it allows direct access and examination of these intra-articular problems within the joint. In addition, arthroscopic repair of the ligamentous structures can provide the initial stability required to prevent postoperative subluxation or re-dislocation. Thus, allowing the possibility of early post-operative rehabilitation, it has the potential benefit of better functional outcomes.

For the treatment of shoulder dislocations, the goal is to prevent further dislocation and to restore a satisfactory range of motion and strength to the limb for life. For most patients with shoulder dislocations, pain and dysfunction severely interfere with activities of daily living. Although some articles show that pain in old shoulder dislocations is very mild and range of motion is quite satisfactory, over time, dysfunction and symptoms can eventually develop, potentially to a degree that is intolerable for the patient (14). Most importantly, the best treatment option for old shoulder dislocations is to prevent acute dislocations from developing into old ones in the first place through prompt and accurate diagnosis. It is imperative that patients at risk of underdiagnosis are given and visual attention and that a thorough whole-body examination is performed when there is a high degree of suspicion. In addition to standard simple imaging, as equipment technology improves and becomes more widely available, CT can be considered to assess the joint more accurately if necessary. In patients with a diagnosis of old shoulder dislocation, a closed manipulative repositioning can still be attempted in the first instance. Although in most cases this is difficult and unsuccessful, closed resetting is still recommended as it can still have a positive effect on improving joint function and relieving symptoms. When this fails, surgical repositioning is recommended. However, when choosing a surgical approach, the presence of comorbidities, the patient's pre-injury functional needs and their expectations must also be taken into account, along with their own skills and experience, such as arthroscopic release of the joint and repair of the glenoid labrum. In some patients, excessive and unnecessary treatment, whether by arthroscopic or open techniques, may cause more harm than good. In some patients of advanced age, a very small number of patients with old shoulder dislocations remain asymptomatic and reasonably functional (19), and this article should remind us that in a very small number of patients, no treatment may be the best treatment.

In conclusion, arthroscopic release and repair is an alternative option for the treatment of old anterior shoulder dislocations. The anterior and inferior joint capsule is the main factor blocking repositioning; the capsule can develop secondary contractures that further block repositioning; and soft tissue balancing can achieve joint stabilisation.

## Data Availability

The original contributions presented in the study are included in the article/Supplementary Material, further inquiries can be directed to the corresponding author/s.
